# Oxidative inhibition of AMPKα2 by 4-hydroxynonenal in human atherosclerotic lesions

**DOI:** 10.1016/j.redox.2026.104314

**Published:** 2026-07-21

**Authors:** Shengyue Li, Xin Zhang, Guocheng Zhang, Huihui Yang, Jie Deng, Ming-Hui Zou

**Affiliations:** a Department of Endocrinology and Metabolism, Tianjin Medical University General Hospital, 154 Anshan Road, Tianjin, 300052, China; b Department of Cardiology, The Second Affiliated Hospital, Xian Jiao Tong University, China

**Keywords:** AMPKα2, 4-HNE, Atherosclerosis, Cysteine oxidation, Endothelial cell

## Abstract

**Background:**

AMP-activated protein kinase (AMPK) is a critical regulator of endothelial function, oxidative stress, and inflammation. Lipid peroxidation products such as 4-hydroxynonenal (4-HNE) accumulate under conditions of oxidative stress and are implicated in vascular dysfunction. However, whether 4-HNE directly reacts with AMPK and alters its activity remains unclear.

**Objectives:**

To explore the effects of 4-HNE on the LKB1-AMPK axis, with a focus on AMPKα1 and AMPKα2 in different cell types (HUVECs, H9c2 cells), a cell-free system, and *in vivo* (Akita mice), and to understand the underlying mechanisms in human atherosclerotic plaques.

**Methods:**

In HUVECs and H9c2 cells, cells were treated with 4-HNE, and protein levels were detected by Western blots. AMPKα2 activity was measured after enrichment via immunoprecipitation. Cell viability was also examined. In a cell-free system, recombinant AMPK was incubated with 4-HNE. In Akita mice, AMPKα2 activity was measured in aortas after tempol treatment. In human atherosclerotic plaques, immunohistochemistry, co - immunoprecipitation, and mass spectrometry were employed.

**Results:**

In HUVECs, low 4-HNE concentrations (1-15 μM) didn't change LKB1 levels, but higher ones (20-25 μM) reduced them. 4-HNE had a variable impact on AMPKα1 levels, while significantly inhibiting AMPKα2 activity in a dose - and time-dependent manner. In H9c2 cells, 4-HNE (10 μM) selectively decreased AMPKα2 levels. In the cell-free system, 4-HNE dose-dependently inhibited AMPKα2 activity. In Akita mice, oxidative stress reduced aortic AMPKα2 activity, reversed by Tempol. In human plaques, 4-HNE levels correlated with plaque severity and AMPKα2 inhibition. Cys174 and Cys200 were key for 4-HNE-mediated AMPKα2 inactivation 4-HNE modified AMPKα2 at Cys174 and Cys200, and antioxidants blocked 4-HNE-induced inhibition. Finally, pretreatment with N-ethylmaleimide, a cysteine blocker, abolished the binding of 4-HNE and AMPKα2, supporting a primary role for cysteine modification in the binding of 4-HNE with AMPKα2.

**Conclusions:**

We conclude that 4-HNE inhibits AMPKα2 activity through oxidative modification of specific cysteine residues in human atherosclerosis.

## Highlights

• 4-HNE inhibits AMPK alpha 2 via Cys174/Cys200 modification in a dose/time-dependent manner. Antioxidants abolish suppression. Pathological 4-HNE accumulation impairs AMPK alpha 2, yielding a vascular disease therapeutic target.

## Graphical abstract

**Figure:**
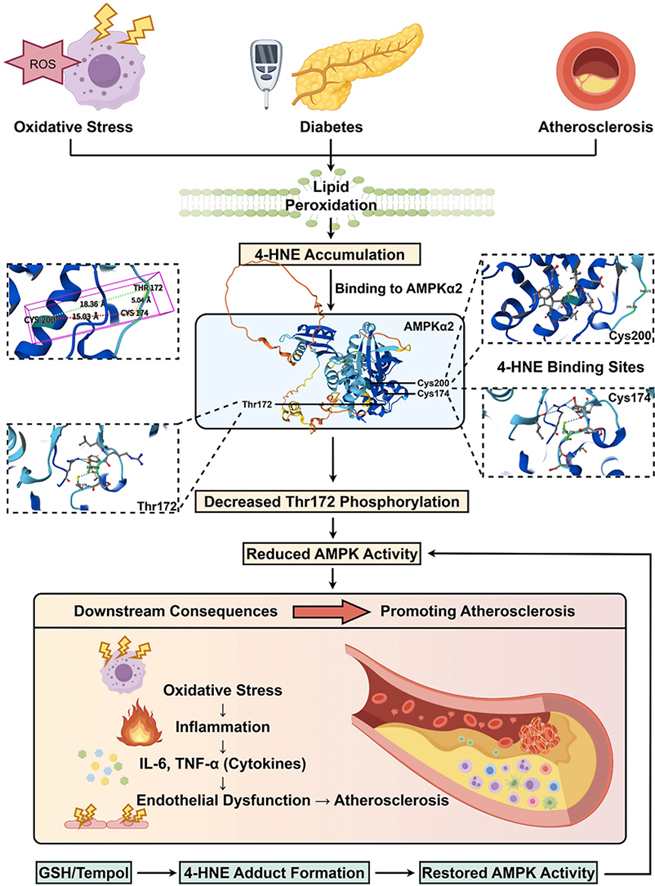
The protein structure in the graphic abstract was open-access predicated by AlphaFold3, and all remaining components were generated using authorized Figdraw resources.

## 1 Introduction

Atherosclerosis is characterized by the accumulation of oxidized lipoproteins in large arteries. Oxidation of lipoprotein yields dozens of lipid products, such as oxysterols, aldehydes, and oxidized fatty acids [1], that may promote the progression of atherosclerosis by stimulating inflammation, foam cell formation, and vascular cell apoptosis [2]. Notably, 4-Hydroxynonenal (4-HNE)—one of the most abundant aldehyde products of lipid peroxidation—is implicated in the pathogenesis of atherosclerosis in humans [3], although its precise role remains unclear.

AMP-activated protein kinase (AMPK) is a serine/threonine protein kinase consisting of a catalytic subunit (α), and two regulatory subunits (β and γ) that have multiple isoforms variants [[4], [5], [6]]. AMPK activity is regulated by allosteric modulation, auto-inhibition, and phosphorylation of the catalytic and regulatory subunits [7], and is stimulated by an increased intracellular AMP:ATP ratio. Allosteric activation of AMPK occurs following binding of AMP to the γ regulatory subunit, which induces a conformational change that prevents AMPK dephosphorylation [7]. Phosphorylation of the catalytic α subunit at Thr172 in the activation loop is essential for significant kinase activity. AMPK is regulated by at least 3 kinases: the serine/threonine kinase LKB1 [[8], [9], [10]], Ca^2+^/calmodulin-dependent protein kinase kinase β (CAMKKβ) [[11], [12], [13], [14]], and the mitogen-activated protein kinase kinase kinase family member TAK1 [15,16]. AMPK phosphorylates several substrates that either regulate energy metabolism [17,18] or exerts multiple protective effects on vascular cells [19,20]. In endothelial cells, phosphorylation of nitric oxide synthase at Ser1177 and Ser633 [21] by AMPKα1 [22] and α2 [23] stimulates nitric oxide production [[21], [22], [23]]. In addition to its effects on endothelial cells, AMPK also influences vascular smooth muscle cells (VSMCs), monocytes, and macrophages [24,25]; For example, the AMPK activator 5-aminoimidazole-4-carboxamide ribonucleotide prevents angiotensin II-stimulated VSMC proliferation [25] and suppresses neointimal formation in response to vascular injury [25].

AMPK also plays a critical role in redox regulation by enhancing antioxidant defenses, including superoxide dismutase (SOD) and catalase, and by activating the Nrf2 pathway, thereby protecting cells from oxidative damage [[26], [27], [28]]. In addition, AMPK supports mitochondrial homeostasis by promoting mitochondrial biogenesis and efficient oxidative phosphorylation, reducing reactive oxygen species (ROS) generated by dysfunctional mitochondria. AMPK further limits oxidative stress by stimulating autophagy, which facilitates the removal of damaged organelles [[29], [30], [31]]. Conversely, AMPK inhibition disrupts redox homeostasis and impairs antioxidant capacity, mitochondrial function, and autophagy resulting in excessive ROS accumulation. These disturbances exacerbate oxidative damage and contribute to the progression of diseases such as atherosclerosis and cancer. In these settings, AMPK inhibition amplifies oxidative stress, vascular injury, and tumor progression [32], underscoring AMPK as a critical therapeutic target in oxidative stress–associated diseases.

4-HNE is a highly electrophilic aldehyde that reacts readily with phospholipids, proteins, and nucleotides [[33], [34], [35]]. Through Michael-type addition reactions, 4-HNE covalently modifies nucleophilic amino acid residues, including histidine, lysine, serine, cysteine, and tyrosine, thereby altering protein structure [36] and impairing enzymatic activity [37]. The reaction kinetics between 4-HNE and cysteine thiolates are extremely rapid, with second-order rate constants of approximately 1000–10,000 M^−1^ s^−1^. Consequently, 4-HNE–cysteine adduct formation occurs within milliseconds to seconds at physiological concentrations and is effectively diffusion-limited *in vivo* [38,39].

In the present study, we investigated the effects of 4-HNE on AMPK activity in cell-free systems, cultured endothelial cells, and *in vivo* models of oxidative stress. We further examined human atherosclerotic tissues to assess the clinical relevance of this mechanism. Finally, we sought to identify the specific amino acid residues within AMPKα2 that mediate its interaction with 4-HNE and determine how this modification influences AMPK activation.

## 2 Materials and methods

### 2.1 Materials

**Chemicals:** Recombinant human AMPKα2β1γ1 heterotrimer protein was purchases from (MedChemExpress MCE, Cat#: HY-P76142). Both 4-HNE (AbMole,Cat# M14296) and N-Ethylmaleimide (NEM, Sigma,Cat# 04260) were dissolved in DMSO. The final DMSO concentration in the medium was 0.05%. Protein A Sepharose CL-48 (Cat# 10-1041) was obtained from Invitrogen. [^32^P]-ATP was purchased from PerkinElmer (Waltham, MA, USA). d-glucose (Cat# G7528), l-glucose (Cat# G5500), glutathione (GSH; Cat# 3541), SAMS peptide (Cat# 12-355), Tempol (Cat# 581500), and other unspecified chemicals were obtained from Sigma-Aldrich (St. Louis, MO, USA) in the highest available grade.

**Cells:** Human umbilical vein endothelial cells (HUVECs) were sourced from Cascade Biologics, Portland, OR, USA). Rat H9c2 cardiomyocytes (CRL-1446) and human embryonic kidney 293 cells (HEK293, CRL-3249) were obtained from the American Type Culture Collection, ATCC, Manassas, VA, USA).

**Plasmids:** The OriGene AMPKα2 plasmid (PRKAA2) (pCMV6-XL4, Cat#: SC323523 Rockville, MD, USA) was employed to overexpress AMPKα2 in HUVECs and HEK293 cells. The OriGene GFP plasmids (pCMV6-AC-GFP, Cat# No: ABIN5371977) were used as a control. The transfection efficiency was >85% in HEK293 cells and approximately 75% in HUVECs.

**Antibodies:** The sources and dilution of antibodies and secondary antibodies and other reagents used in this study have been listed in Supplemental Table 1.

### 2.2 Human subjects

Human left and right coronary arterial specimens were obtained from eight post-mortem donated bodies (four men and four women) with institutional review board approval. The protocol received approval from the Ethics Committee of the Second Affiliated Hospital of Xi'an Jiaotong University (IRB2025-241) and was conducted in accordance with the Declaration of Helsinki. Atherosclerotic lesions were stratified into three progressive stages according to luminal stenosis degree (10%, 40%, and 90% narrowing) and histological verification. Total proteins were extracted from staged plaque tissues via liquid nitrogen pulverization and RIPA lysis with inhibitor supplementation, followed by quantitative protein detection. Immunocytohistochemical staining with specific primary and secondary antibodies was performed on graded plaque sections to detect targeted protein expression and localization.

### 2.3 Animals

Male Akita and wild - type (WT) C57BL/6 mice (5 weeks old; sourced from Jackson Laboratory, Bar Harbor, ME, USA) were housed under specific pathogen-free conditions at Tianjin Medical University. Environmental parameters, including temperature, humidity, and a 12-h light-dark cycle, were stringently controlled. The mice had unrestricted access to sterilized water and standard chow.

Tempol, a superoxide dismutase mimetic that suppresses oxidant formation essential for 4-HNE generation, was employed to evaluate the contribution of endogenous oxidants to AMPK oxidation and inhibition. WT and Akita mice were randomly assigned to receive either Tempol (1 mM) in drinking water or plain water for an 8-week period.

Blood glucose levels were measured in tail blood using the Reli - On Ultima Blood Glucose Monitoring System. Subsequently, the mice were euthanized, and their aortas were promptly harvested and frozen in liquid nitrogen for subsequent AMPK activity analysis. All animal procedures were approved by the Institutional Animal Care and Use Committee of Tianjin Medical University.

### 2.4 Cell culture and treatments

#### 2.4.1 HEK293 cells

HEK293 cells were maintained in Dulbecco's Modified Eagle Medium (DMEM) supplemented with 10% FBS. HUVECs were cultured in Medium 200 supplemented with low - serum growth supplements and 10% FBS until complete confluency was achieved.

#### 2.4.2 H9c2 cells

The rat myocardial cell line H9c2 was cultured in DMEM (DMEM/High, BasalMedia, Shanghai) containing 10% fetal bovine serum (FBS, F101-01, Vazyme) and supplemented with Penicillin - Streptomycin (15140122, Gibco). The cells were incubated in a humidified atmosphere with 5% CO_2_ at 37°C. H9c2 cells were seeded into 6-well plates and reached approximately 90% confluence the following day. The cells were then serum - starved in 1% FBS for 6 h, after which the medium was replaced with serum-free DMEM and 4-HNE was added to a final concentration of 10 μM for 30 min. If continued culture was required, 2% FBS was replenished after the 30-min incubation.

H9c2 cells were seeded into 6-well plates and reached approximately 90% confluence the following day. After serum starvation in 1% FBS for 6 h, the cells were pretreated with NEM (25 or 50 μM) for 30 min. Subsequently, the medium was replaced with serum-free DMEM, and 4-HNE (10 μM) along with NEM (25 μM) was added for 30 min. The final DMSO concentration in the medium was 0.1%.

#### 2.4.3 HUVECs

Upon attaining confluency in 6-well plates, HUVECs were serum - starved in medium containing 1% FBS for 12 h, followed by incubation in serum - free DMEM for 30 min. The cells were then treated with 4-HNE at the indicated concentrations for 30 min. After 4-HNE treatment, the cells were incubated in medium containing 2% FBS for the specified durations. To assess the effects of reducing agents on AMPKα2 suppression induced by 4-HNE or high glucose (HG, 25 mM d-glucose). 19.5 mM l-glucose plus 5.5 mM d-glucose are used as osmotic control., HUVECs were pretreated with GSH (5 mM) for 1 h, followed by treatment with either 4-HNE or HG for the indicated times.

#### 2.4.4 Plasmid construction and gene transfer

The OriGene AMPKα2 plasmid (PRKAA2) (RG210226) was utilized. Each of the eight cysteine residues (Cys106, Cys130, Cys174, Cys200, Cys209, Cys297, Cys302, and Cys382) was individually mutated to alanine using the QuikChange site-directed mutagenesis kit (Stratagene), in accordance with the manufacturer's instructions. The primers for AMPK mutation are shown in Table 1. All mutations were verified by DNA sequencing. Plasmid DNA was purified using the Qiagen EndoFree Plasmid Maxi Kit. HEK293 cells were transfected with wild-type or mutant AMPKα2 plasmids using Lipofectamine 2000 (Invitrogen, Cat# 11668500), as previously described [40]. After 24 h, the cells were treated with 4-HNE for 4 h and then harvested for AMPK activity assays.

**Table 1:** Primers used for AMPK mutation.

Primer Name	Sequence
cysteine106-sense	5-ttatttgactacatcgctaagcatggacgggtt-3
cysteine106-antisense	5-aacccgtccatgcttagcgatgtagtcaaataa-3
cysteine130-sense	5-tctgctgtggattacgctcataggcatatggtt-3
cysteine130-antisense	5-aaccatatgcctatgagcgtaatccacagcaga-3
cysteine174-sense	5-tttctgagaactagtgccggatctccaaattat-3
cysteine174-antisense	5-ataatttggagatccggcactagttctcagaaa-3
cysteine200-sense	5-gttgatatctggagcgctggtgttatcttgtat-3
cysteine200-antisense	5-atacaagataacaccagcgctccagatatcaac-3
cysteine209-sense	5-ttgtatgctcttcttgctggcaccctcccattt-3
cysteine209-antisense	5-aaatgggagggtgccagcaagaagagcatacaa-3
cysteine297-sense	5-gctgtgaaagaagtggctgaaaaatttgaatgt-3
cysteine297-antisense	5-acattcaaatttttcagccacttctttcacagc-3
cysteine302-sense	5-tgtgaaaaatttgaagctacagaatcagaagta-3
cysteine302-antisense	5-tacttctgattctgtagcttcaaatttttcaca-3
cysteine382-sense	5-agccccaaagcaagagctccattggatgcactg-3
cysteine382-antisense	5-cagtgcatccaatggagctcttgctttggggct-3

#### 2.4.5 Assays of 4-HNE on recombinant AMPKα2β1γ1

The inhibitory effect of 4-HNE (10 μM) on recombinant AMPKα2β1γ1 was evaluated at 5, 10, 30, 60, and 120 min. A dose - response curve was generated following a 120 - minute incubation with 1, 5, 10, 50, or 100 μM 4-HNE. AMPKα2 activity was measured using the SAMS peptide and [^32^P]-ATP, as described previously. To determine the effect of GSH, recombinant AMPKα2β1γ1 was incubated with GSH (5 mM) for 60 min, followed by the addition of 4-HNE (10 μM) for an additional 120 min.

#### 2.4.6 Immunoprecipitation of AMPKα2

Equal quantities of protein from human coronary arteries with atherosclerosis, heart tissues from Akita mice, or HUVECs were lysed in cold RIPA buffer (comprising Tris-HCl, NaCl, sodium dodecyl sulfate (SDS), sodium deoxycholate, and Triton X-100) and incubated on ice for 20 min in the presence of protease inhibitors. The supernatant was incubated with 10 μL (1:100 dilution) of nonspecific mouse IgG (Cell Signaling Technology) for 1 h at 4°C, followed by incubation with 50 μL protein A/G agarose beads (Thermo Scientific™,Cat# No. 20421) for 1 h at 4°C for pre-clearing. The cleared lysate was then incubated with anti - AMPKα2 antibody (1:100 dilution; Cat# sc - 19131, Santa Cruz Biotechnology) overnight at 4°C. Subsequently, 50 μL protein A/G agarose beads were added to capture AMPKα2 immune complexes. The beads were washed, and the bound proteins were eluted in 50 μL SDS-PAGE buffer by boiling at 100°C for 5 min.

#### 2.4.7 Western blot analysis

Protein lysates from human coronary arteries, mouse aortas, or HUVECs, as well as immunoprecipitated proteins from agarose beads (50 μL), were mixed with equal volumes of SDS-PAGE buffer and heated at 100°C for 5 min. Proteins were separated by sodium dodecyl sulfate-polyacrylamide gel electrophoresis (SDS - PAGE) and transferred to nitrocellulose membranes. The membranes were blocked with 4% non - fat milk (Sigma - Aldrich) for 2 h and incubated with primary antibodies against AMPKα2, 4-HNE, phospho-AMPK (Thr172), phospho-ACC (Ser79), or total ACC. The membranes were then incubated with secondary antibodies (1:5000 dilution) for 1 h and developed using enhanced chemiluminescence (Pierce), as described previously [41,42]. Band intensities were quantified using an MSF-300G scanner (Microtek/Nikon).

#### 2.4.8 In vitro AMPKα2 kinase assay

Immunoprecipitated proteins or purified AMPK were incubated with 60 μL agarose beads for 3 h. The kinase reaction mixture consisted of 5 μL of 10× kinase buffer containing dithiothreitol (DTT), 10 μL SAMS peptide, 10 μL ATP working stock (prepared from 100 mM ATP, 0.1 μL [^32^P]-ATP, and 9.8 μL H_2_O), and 25 μL adenosine monophosphate (AMP). The agarose beads were incubated with 50 μL of the reaction mixture for 15 min. Subsequently, 25 μL of the supernatant was spotted onto P81 phosphocellulose paper, which was washed five times with 1% phosphoric acid. The paper was then dried and placed into scintillation vials for radioactivity counting (counts per minute, cpm), as previously described.

#### 2.4.9 Immunohistochemistry and immunofluorescence

Human coronary arteries were obtained within 24 h post - traumatic death. Normal arteries were defined by the absence of morphological features characteristic of atherosclerosis. Paraffin-embedded sections from the right and left coronary arteries were prepared. The slides were boiled in citrate buffer (pH 6.0), allowed to cool spontaneously, and washed three times with phosphate - buffered saline (PBS). Following antigen retrieval (citrate buffer, pH 6.0, 100°C for 10 min), the sections were incubated overnight at 4°C with primary antibodies against AMPKα2, 4-HNE, or phospho-AMPK (Thr172). After washing, the sections were incubated with horseradish peroxidase (HRP)-conjugated secondary antibodies. HRP activity was visualized using 3,3′-diaminobenzidine (DAB). For immunofluorescence, the sections were incubated with fluorescently labeled secondary antibodies for 30 min, as previously described. Images were acquired using a light or confocal microscope, and integrated optical densities were quantified using Image-Pro Plus 5.1 software.

#### 2.4.10 Mass spectrometry

Recombinant AMPKα2β1γ1 was resuspended in a buffer containing 50 mM Tris-HCl and 2 mM reduced GSH (pH 8.0). 4-HNE was added to a final concentration of 1 mM to 50 μL of the AMPKα2 solution (2.5 μg) and incubated at room temperature for 5 h. Sequencing - grade trypsin was added at an enzyme - to - protein ratio of 1:50, and the mixture was incubated overnight at 37°C. To enhance the coverage of potential modification sites, thermolysin digestion was also carried out using a buffer containing 50 mM Tris - HCl and 0.5 mM CaCl_2_ at 70–90°C for approximately 5 h.

The digested peptides were concentrated to 20 μL by vacuum centrifugation and desalted using C18 ZipTip columns. The samples were analyzed using a 4800 MALDI-TOF/TOF mass spectrometer (ABI, Foster City, CA, USA), with mass tolerances of 0.15 Da for peptide mass fingerprinting (PMF) and 0.25 Da for tandem mass spectrometry (MS/MS) [43]. Protein identification was performed using GPS Explorer software, with a confidence threshold greater than 99.99%. The sites of 4-HNE modification on AMPKα2 were identified based on Mascot database searches and manual interpretation of MS/MS spectra.

#### 2.4.11 Statistical analysis

Data are presented as the mean ± standard error of the mean (SEM). The normality of data distribution was evaluated using the Shapiro‒Wilk test, and the equality of variances was determined by the F test or Brown-Forsythe test. For comparisons between two groups, unpaired Student's t-tests were utilized for normally distributed data; otherwise, the non - parametric Mann‒Whitney *U* test was employed. In cases where variances were unequal, the Welch *t*-test was applied. For comparisons among more than two groups, one-way analysis of variance (ANOVA) followed by Tukey's post-hoc test was used if the data passed the equal-variance test (Brown-Forsythe test). If variances were unequal, Welch ANOVA with Dunnett's T3 post - hoc test was employed. For non-normally distributed data, the Kruskal-Wallis test was utilized. Analyses involving two independent variables were conducted using two-way ANOVA followed by Bonferroni post-hoc test. Associations between categorical variables (e.g., sex, history of coronary heart disease) were analyzed using the Chi-squared (χ^2^) test. Multivariable linear regression analysis was employed to adjust for potential confounders, as detailed in the relevant results section. All statistical analyses were conducted using Stata version 18.0 (StataCorp, Texas) or GraphPad Prism 10.0 (California).

## 3 Results

### 3.1 Effects of 4-HNE on AMPKα1 in HUVECs

**Fig. 1:**
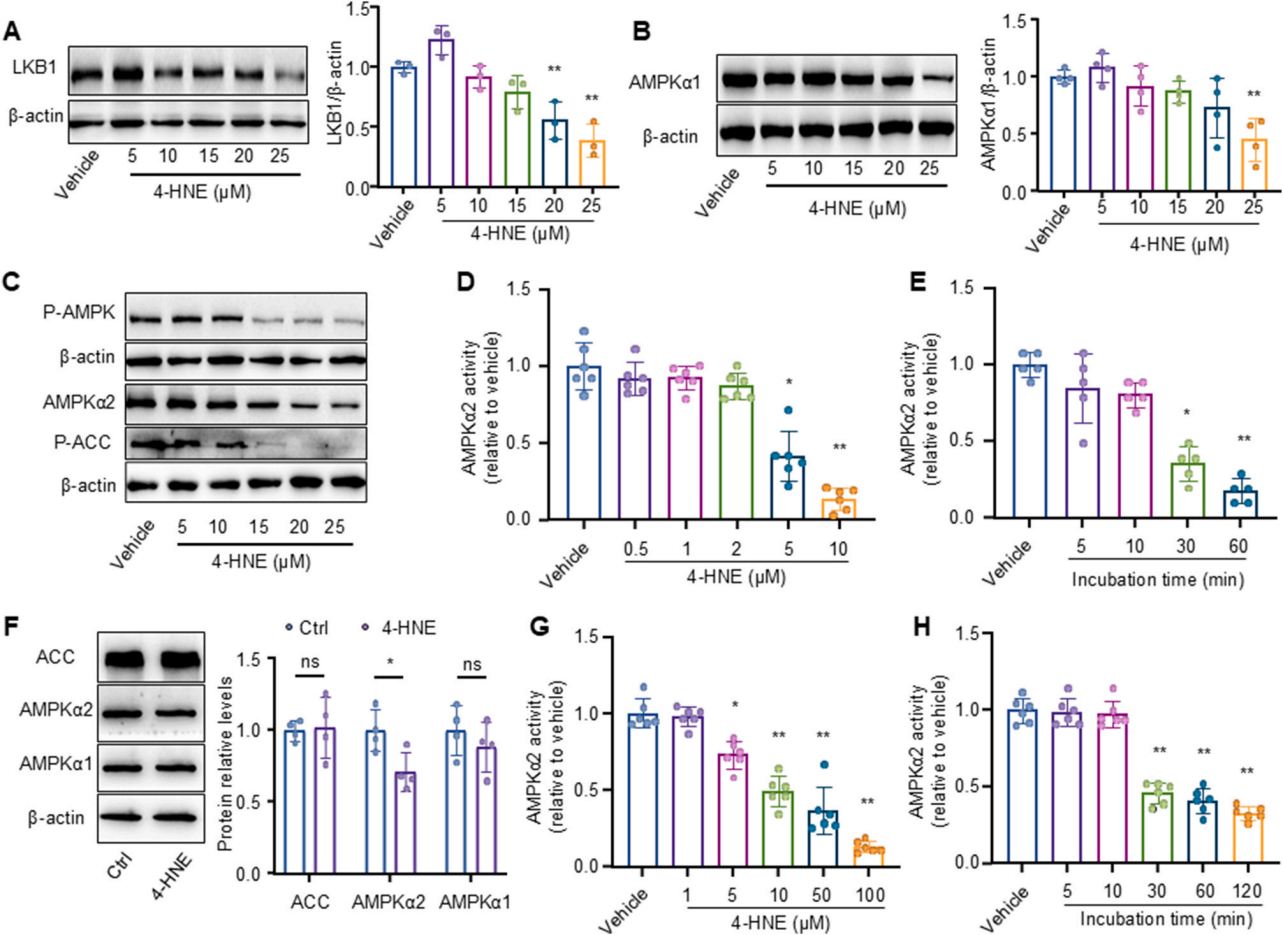
Dose- and time-dependent profiles of 4-HNE-triggered AMPKα2 suppression in recombinant AMPKα2β1γ1 protein, human umbilical vein endothelial cells (HUVECs), and H9c2 cardiomyocytes. (A) Regulatory effects of 4-HNE on LKB1 expression in HUVECs. Confluent HUVECs were subjected to 5–25 μM 4-HNE treatment for 12 h. The expression levels of LKB1 were quantified via Western blot analysis using a specific LKB1 antibody. n = 3, *p < 0.05. (B) Modulatory effects of 4-HNE on AMPKα1 expression in HUVECs. Confluent HUVECs were incubated with 5–25 μM 4-HNE for 12 h. The abundance of AMPKα1 was measured by Western blot assay using an AMPKα1-specific antibody. n = 4, *p < 0.05. (C) Impacts of 4-HNE on the expression of AMPKα2, phosphorylated AMPK (p-AMPK), and phosphorylated ACC (p-ACC) in HUVECs. Confluent HUVECs were exposed to 5–25 μM 4-HNE for 12 h. The protein levels of AMPKα2, p-AMPK, and p-ACC were separately detected through Western blot analysis with corresponding specific antibodies. (D) Dose-response characteristics of AMPKα2 activity in 4-HNE-treated HUVECs. AMPK enzymatic activity was assessed in HUVECs treated with 4-HNE (0.5 to 10 μM) over predefined time intervals. The basal activity of AMPKα2 in HUVEC is 0.102 nmol/min/mg proteins of whole HUVECs lysates. n = 6, *p < 0.05, **p < 0.01. (E) Time-course profile of 4-HNE-induced AMPK suppression in HUVECs. HUVECs were incubated with 10 μM 4-HNE for designated durations. n = 5, *p < 0.05, **p < 0.01. (F) Effects of 4-HNE on AMPKα2, p-AMPK, and p-ACC expression in H9c2 cells. H9c2 cells were treated with 10 μM 4-HNE for 30 min, and the protein levels of AMPKα1, AMPKα2, and ACC were quantified via Western blot analysis using their respective specific antibodies. n = 4, *p < 0.05. (**G**) In vitro dose-dependent suppression of AMPKα2 activity by 4HNE. Partially purified recombinant AMPKα2β1γ1 complexes were incubated with gradient concentrations of 4-HNE (1 to 100 μK) for 2 h. AMPK enzymatic activity was evaluated by detecting [^32^P]-ATP incorporation into SAMS peptides. The basal activity of AMPKα2 in recombinant AMPKα2β1γ1 was 87.667 nmol/min/mg proteins. n = 6, *p < 0.05, **p < 0.01. (**H**) In vitro time-dependent inhibition of AMPKα2 activity by 4-HNE. Partially purified recombinant AMPKα2β1γ1 complexes were treated with 10 μM 4-HNE for time points indicated. AMPKα2 enzymatic activity was determined via the [^32^P]-ATP incorporation assay targeting SAMS peptides. n = 6, *p < 0.05, **p < 0.01.

Our previous work [32, 41] demonstrated that AMPKα1 is the dominant isoform (>80%) in endothelial cells and that LKB1 (liver kinase B1) is a main upstream kinase of AMPK. To examine the effects of 4-HNE on the LKB1-AMPK axis, confluent HUVECs were treated with 4-HNE at concentrations ranging from 5 to 25 μM for 12 h. Protein extracts were then probed for LKB1, AMPKα1, AMPKα2, p-AMPKα (Thr172), and p-ACC (Ser79) using specific antibodies by Western blotting. As shown in Fig. 1A, low concentrations of 4-HNE (5-15 μM) did not significantly alter LKB1 levels, whereas higher concentrations (20-25 μM) significantly reduced LKB1 levels in HUVECs. Similarly, exposure to 4-HNE at 5-20 μM had no significant effect on AMPKα1, although a high concentration (25 μM) significantly lowered the AMPKα1 levels (Fig. 1B). In contrast, AMPKα2 levels in 4-HNE-treated HUVECs were markedly lower than those in vehicle-treated counterparts (Fig. 1C). Consistently, the levels of p-AMPK and p-ACC, a well - characterized downstream target of AMPK, were dose-dependently suppressed by 4-HNE treatment (Fig. 1C).

### 3.2 4-HNE inhibits AMPKα2 activity in HUVECs

As AMPKα2 is a minor isoform of AMPKα with relatively low expression levels, we first enriched AMPKα2 by immunoprecipitation using an AMPKα2-specific antibody. We then measured AMPKα2 activity by assaying the phosphorylation of SAMS peptides using ^32^P - ATP. As shown in Fig. 1D and E, 4-HNE inhibited AMPKα2 activity in a dose- and time-dependent manner. AMPKα2 activity was significantly reduced by 5 μM 4-HNE and further declined at 10 μM (Fig. 1D). Consistent with the in vitro findings, AMPKα2 activity was significantly inhibited 30 min after exposure to 4-HNE (10 μM) and was almost completely inhibited after 60 min (Fig. 1E).

### 3.3 Effects of 4-HNE on cell viability in HUVECs

We then assessed the effect of 4-HNE on HUVEC viability. As shown in Supplementary Fig. 1, exposure to 5 μM 4-HNE for 24 h did not significantly affect cell viability, whereas higher concentrations (>10 μM) markedly decreased viability. Together with the observation that 4-HNE suppresses AMPK activity at concentrations as low as 5 μM (Fig. 1C), these results indicate that low concentrations of 4-HNE (≤5 μM) inhibit AMPK without causing cytotoxicity.

### 3.4 4-HNE inhibits AMPKα2 activity in H9c2 cells

Unlike HUVECs, cardiomyocytes express both AMPKα1 and AMPKα2, with AMPKα2 being the dominant isoform of AMPKα. To examine the selectivity of 4-HNE for AMPKα2, H9c2 cells were treated with 10 μM 4-HNE for 12 h. As shown in Figs. 1F and 4-HNE (10 μM) significantly lowered the levels of AMPKα2 but had no effect on AMPKα1, confirming a higher reactivity of AMPKα2 than AMPKα1 towards 4-HNE.

### 3.5 4-HNE inhibits AMPKα2 activity in a cell - free system

We next determined whether 4-HNE directly inhibits AMPK activity in vitro. Purified recombinant α2/β1/γ1 AMPK (25 ng) was incubated at room temperature with 4-HNE (1-100 μM) for 120 min. As shown in Figs. 1G and 4-HNE dose-dependently inhibited AMPKα2 activity from 5 to 100 μM. Maximal inhibition (>80%) was observed at 100 μM 4-HNE (Fig. 1G). Additionally, purified AMPKα2 protein was incubated with 4-HNE (5 μM) for 5-120 min. As shown in Fig. 1H, AMPKα2 kinase activity was significantly reduced 30 min after 4-HNE addition, and inhibition persisted throughout the 120- minute study period.

### 3.6 AMPK activity is suppressed in Akita mouse aortas

Diabetes is a well - established risk factor for atherosclerosis. Patients with type I diabetes have a high risk of developing cardiovascular diseases, including atherosclerosis. Oxidative stress plays an important role in the pathogenesis of diabetic complications, and 4-HNE is known to accumulate in diabetic animals. At 13 weeks of age, Akita mice, a well-established model of type I diabetes, exhibited significantly lower body weight (Supplementary Fig. 2A) and elevated blood glucose levels (Supplementary Fig. 2B) compared with wild type (WT) controls. Tempol, a potent antioxidant, did not normalize body weight or blood glucose levels in Akita mice (Supplementary Fig. 2A and 2B).

**Fig. 2:**
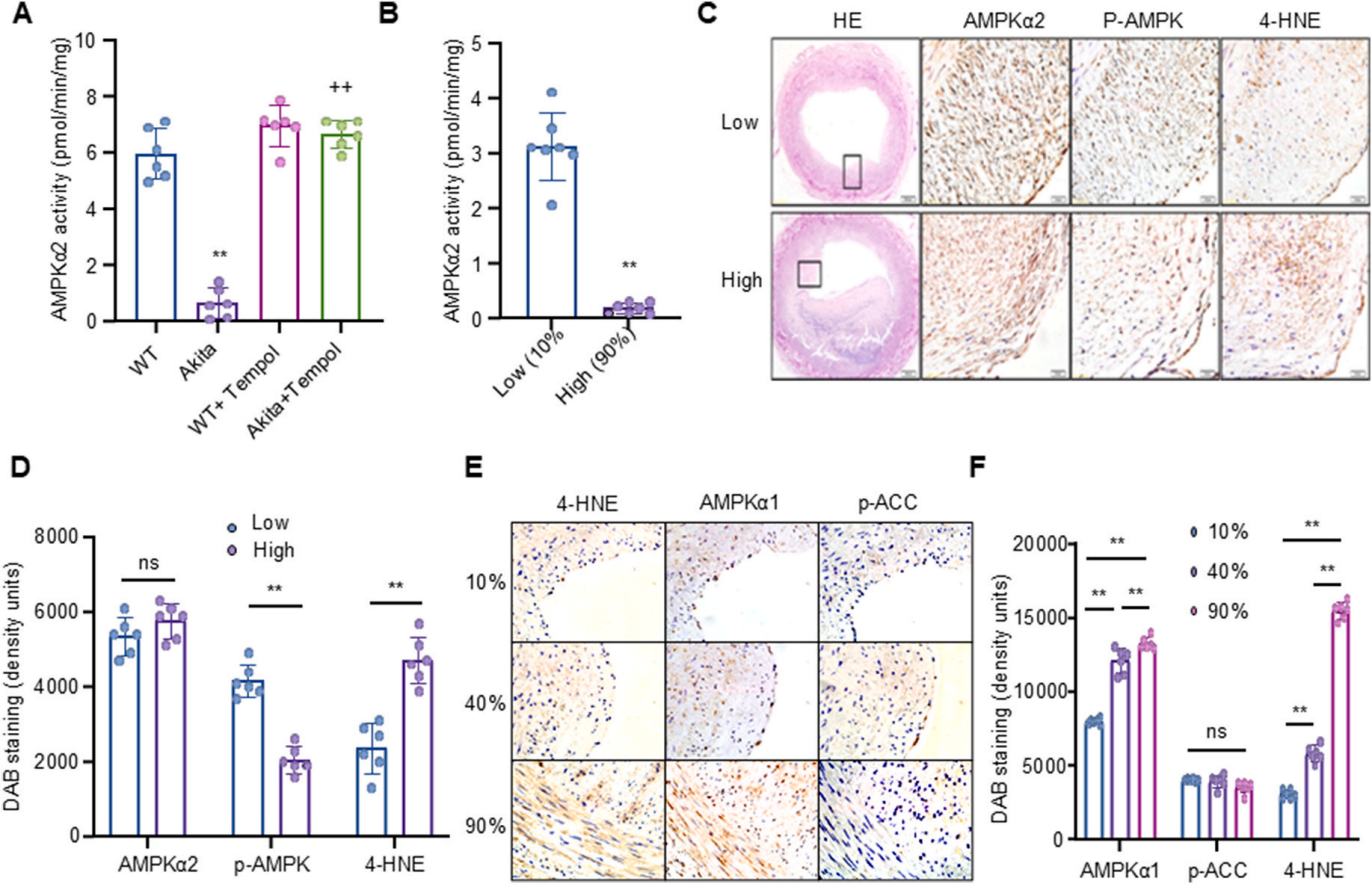
AMPK enzymatic activity is downregulated in aortic tissues of diabetic mice and human atherosclerotic lesions. (**A**) Alterations in aortic AMPK activity in wild-type (WT) and Akita diabetic mice following tempol intervention. WT and Akita diabetic mice were administered with 1 mM tempol via drinking water or vehicle solution for an 8-week intervention, after which aortic AMPK enzymatic activity was detected. n = 6, **p < 0.01 for Akita group versus control group; ^++^*p* < 0.01 for Akita group versus Akita + tempol group. (**B**) AMPK activity profiling in human coronary arterial tissues. The relative AMPK enzymatic activity in human coronary artery specimens was quantified for each experimental group (n = 7 per group). **p < 0.01 versus the control group. (**C** & **D**) Downregulated expression of p-AMPK along with increased levels of 4-HNE staining in advanced human atherosclerotic lesions. Immunohistochemical staining of 4-HNE, AMPKα2, and p-AMPK was performed on human atherosclerotic lesion tissues using corresponding specific antibodies with 3,3′-diaminobenzidine (DAB) as the chromogenic substrate. Tissue sections were visualized and analyzed via confocal microscopy, and positive staining areas were quantified using Image-Pro Plus 5.1 software. n = 6 per group, *p < 0.05, **p < 0.01 versus the control group. (**E** & **F**) Representative immunohistochemical (DAB) staining of 4-HNE, AMPKα1 and p-ACC in human atherosclerotic lesions with different plaque coverage. Immunostaining assays were conducted on human atherosclerotic lesions with luminal plaque coverage rates of 10%, 40%, and 90% and tissue sections were visualized and analyzed via confocal microscopy, and positive staining areas were quantified using Image-Pro Plus 5.1 software. n = 6, **p < 0.01.

AMPKα2 activity was measured in WT and Akita mouse aortas after 8 weeks of tempol exposure. Compared with WT mice, unexposed Akita mice exhibited reduced AMPKα2 activity (Fig. 2A). In contrast, AMPKα2 activity in tempol-treated Akita mouse aortas was comparable to that in WT mice (Fig. 2A).

### 3.7 Reduced AMPKα2 activity in human atherosclerotic plaques

4-HNE is implicated in the pathogenesis of atherosclerosis. To determine whether 4-HNE accelerates atherosclerosis through inhibition of AMPKα2, we measured AMPKα2 activity in post-mortem coronary artery samples from plaque-free regions and atherosclerotic plaques. Compared with atherosclerotic lesion low coverage arteries, (10%), AMPKα2 activity was significantly reduced in coronary arteries from the arteries with atherosclerotic high area (90%) patients (Fig. 2B), consistent with a role for AMPK inhibition in human atherosclerosis.

### 3.8 Reduced AMPKα2 phosphorylation is associated with elevated 4-HNE levels in human atherosclerotic plaques

We further analyzed the relationship between 4-HNE and AMPKα2 in human atherosclerotic plaques using immunohistochemistry. As shown in Fig. 2C, strong staining for AMPKα2, phospho-AMPKα (Thr172) and 4-HNE. All vascular cell types were positive for AMPK, phospho-AMPKα (Thr172), and 4-HNE, with the strongest staining detected in the endothelium (Fig. 2C). Although AMPKα2 levels in atherosclerotic plaques were comparable to those in normal coronary arteries, p-AMPKα immunostaining was significantly reduced in high atherosclerotic lesion samples (Fig. 2C and D), indicating an inhibition of AMPKα2 in atherosclerotic lesions.

Next, we assayed the levels of 4-HNE, AMPKα1, and p-ACC in human coronary atherosclerotic plaques with varying plaque coverage (10%, 40%, or 90% of the luminal surface). The levels of 4-HNE were significantly elevated as plaque coverage increased from 10 to 90%, while the levels of ACC remained unchanged (Fig. 2E and F). In parallel, the levels of AMPKα1 were elevated with increasing plaque coverage. In contrast, the levels of p-ACC remained unchanged despite the increase in AMPKα1 in the atherosclerotic plaques.

### 3.9 Antioxidants prevent 4-HNE–induced AMPKα2 inhibition

**Fig. 3:**
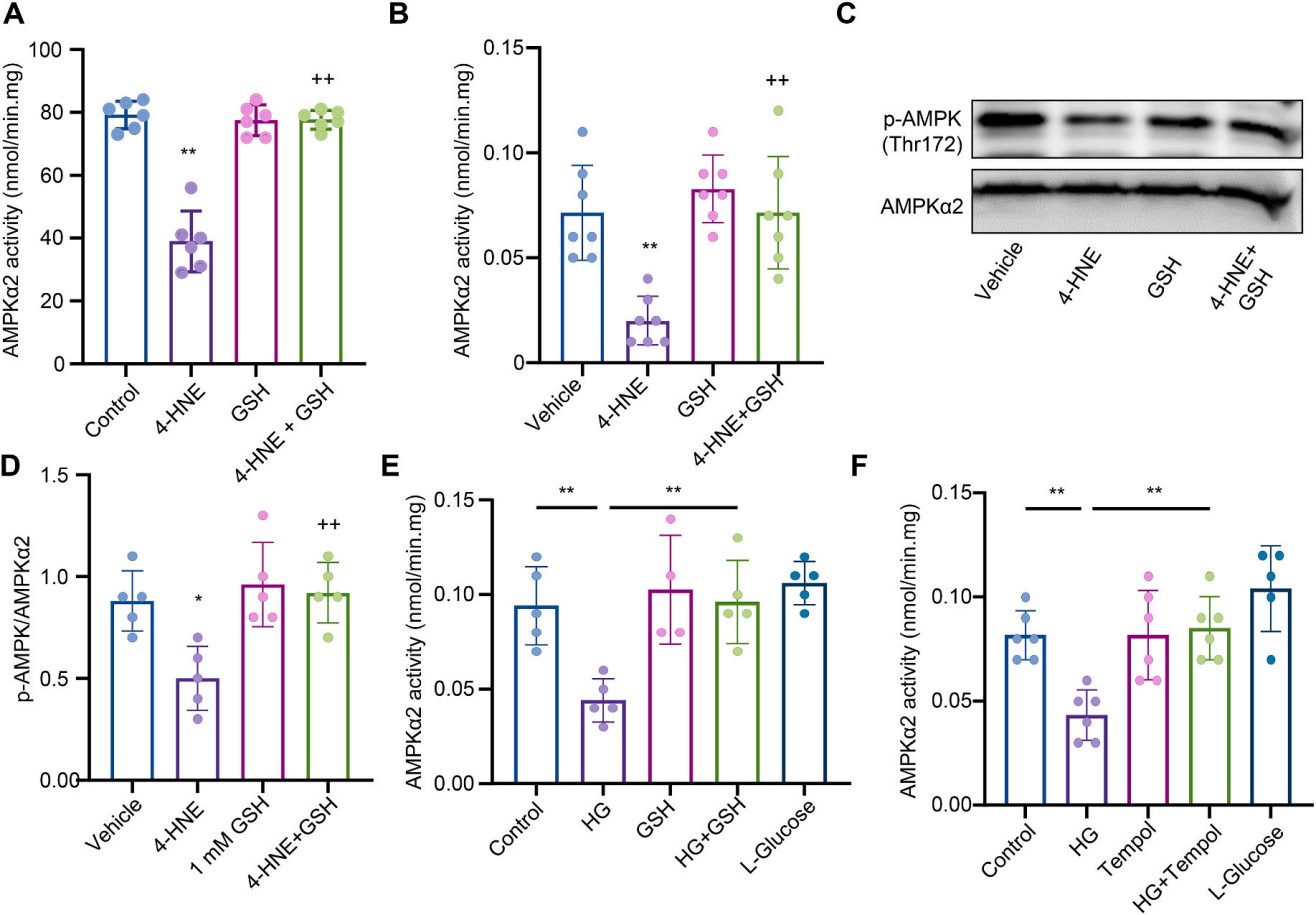
GSH and tempol supplementation attenuates 4-HNE-mediated AMPKα2 inhibition. **(A)** In vitro AMPKα2 activity analysis. Purified AMPKα2 protein was incubated with 10 μM 4-HNE for 30 min in the presence or absence of 2.5 mM GSH. n = 6, **p < 0.01 for 4-HNE group versus control group; ^++^*p* < 0.01 for 4-HNE group versus 4-HNE + GSH co-treatment group. **(B)** AMPKα2 activity in GSH-preconditioned HUVECs upon 4-HNE stimulation. HUVECs were pretreated with 5 mM GSH for 30 min, followed by co-treatment with 5 mM GSH and 5 μM 4-HNE for 4 h n = 7, **p < 0.01 for 4-HNE group versus control group; ^++^*p* < 0.01 for 4-HNE group versus 4-HNE + GSH co-treatment group. **(C)** Western blot detection of AMPK phosphorylation in 4-HNE and GSH co-treated HUVECs. Western blot assays were performed to examine the phosphorylation level of AMPK in HUVECs subjected to single 4-HNE treatment or combined 4-HNE and GSH treatment. **(D)** Densitometric quantification of phosphorylated AMPK protein. The relative expression of phosphorylated AMPK was quantitatively analyzed via densitometry. n = 5, **p < 0.01 for 4-HNE group versus control group and ^++^*p* < 0.01 for 4-HNE group versus 4-HNE + GSH co-treatment group. **(E)** Regulatory effect of GSH on HG-suppressed AMPK activity in HUVECs. HUVECs were cultured under 25 mM HG condition with or without 5 mM GSH intervention for 24 h n = 5, **p < 0.01. **(F)** Rescue effect of tempol on HG-induced AMPK activity reduction in HUVECs. HUVECs were exposed to 25 mM HG medium with or without 100 μM tempol treatment for 24 h n = 6, **p < 0.01.

Oxidative stress can alter protein structure and function through oxidation. Since GSH is an antioxidant that neutralizes reactive oxygen species and scavenges α,β-unsaturated aldehydes, it was used to block 4-HNE adduct formation. To determine whether 4-HNE inhibits AMPKα2 through oxidative mechanisms, we examined the inhibition of AMPKα2 by 4-HNE (10 μM) in the presence of tempol (100 μM) or GSH (5 mM). GSH abolished the inhibitory effect of 4-HNE in both a cell-free system (Fig. 3A) and HUVECs (Fig. 3B). Consistent with the inhibitory effect of 4-HNE on AMPK, the reduction in AMPKα Thr172 phosphorylation induced by 4-HNE was attenuated by the simultaneous addition of GSH (Fig. 3C and D).

**Fig. 4:**
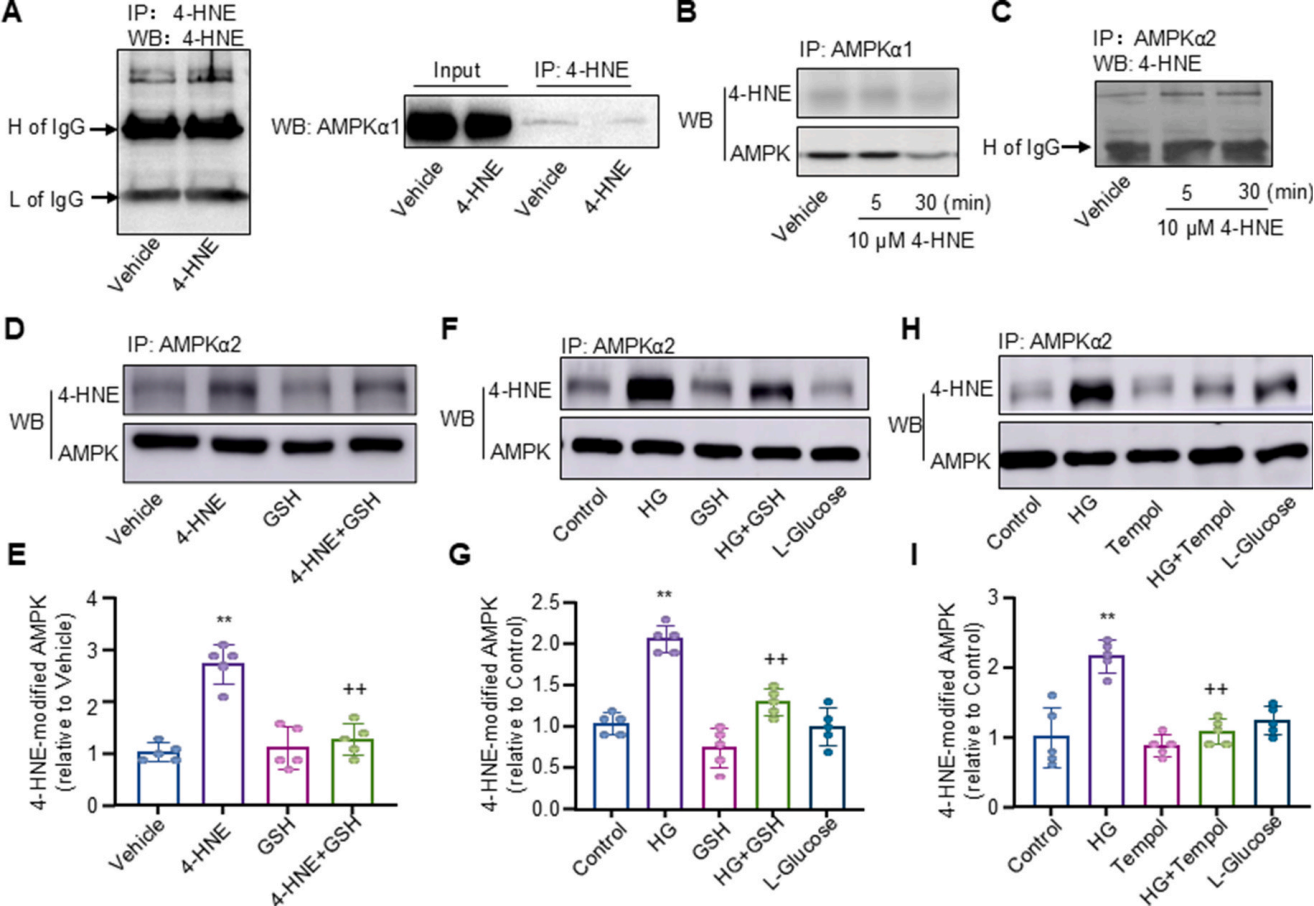
GSH and tempol disrupt the molecular interaction between 4-HNE and AMPKα2. For all relevant experimental assays, 4-HNE, AMPKα1, or AMPKα2 protein was immunoprecipitated from total cellular protein lysates using corresponding specific antibodies. Subsequent Western blot analysis was performed to detect the presence of 4-HNE, AMPKα1, or AMPKα2 within the immunoprecipitated complexes. (**A**) Representative immunoblot images verifying 4-HNE conjugation with cellular proteins but not AMPKα1. Confluent HUVECs were stimulated with 5 μM 4-HNE for 12 h. 4-HNE-conjugated cellular proteins were enriched via immunoprecipitation using a 4-HNE-specific antibody. A distinct 4-HNE-targeted antibody was utilized to validate 4-HNE-bound proteins, and an AMPKα1-specific antibody was applied to detect endogenous AMPKα1 in the 4-HNE immunoprecipitation complexes. (**B**) 4-HNE stimulation fails to induce covalent binding between 4-HNE and AMPKα1. Confluent HUVECs were treated with 10 μM 4-HNE for 5 min or 30 min. AMPKα1 protein was first immunoprecipitated, and Western blot assays were subsequently conducted to assess 4-HNE conjugation to AMPKα1 using a 4-HNE-specific antibody. (**C**) 4-HNE treatment promotes time-dependent covalent binding of 4-HNE to AMPKα2. Confluent HUVECs were incubated with 10 μM 4-HNE for 5 min or 30 min. AMPKα2 was immunoprecipitated, and 4-HNE-bound AMPKα2 was detected by Western blot analysis with a 4-HNE-specific antibody. (**D & E**) GSH eliminates 4-HNE-mediated AMPKα2 modification in HUVECs. HUVECs were pretreated with 5 mM GSH for 30 min prior to 24 h of 5 μM 4-HNE stimulation. AMPKα2 protein was immunoprecipitated, and 4-HNE-conjugated AMPKα2 was examined via Western blot using a 4-HNE-specific antibody. n = 5, **p < 0.01 versus vehicle group; ^++^*p* < 0.01 for 4-HNE monotherapy group versus 4-HNE plus GSH co-treatment group. (**F** & **G**) GSH abolishes high-glucose-induced 4-HNE conjugation to AMPKα2. Confluent HUVECs were cultured for 72 h under high-glucose conditions (25 mM d-glucose) or isotonic control conditions (19.5 mM l-glucose plus 5.5 mM d-glucose), with or without 5 mM GSH supplementation. AMPKα2 was immunoprecipitated using an AMPKα2-specific antibody, and 4-HNE-bound AMPKα2 was detected via Western blot with a 4-HNE-specific antibody. n = 5, **p < 0.01 versus the control group; ^++^*p* < 0.01 for HG group versus HG plus GSH co-treatment group. (**H** & **I**) Tempol attenuates high-glucose-enhanced 4-HNE-AMPKα2 binding in HUVECs. HUVECs were incubated for 72 h in high-glucose medium (25 mM d-glucose) or isotonic control medium (19.5 mM l-glucose plus 5.5 mM d-glucose) with or without 100 μM tempol treatment. AMPKα2 protein was immunoprecipitated, and 4-HNE-conjugated AMPKα2 was quantified via Western blot densitometry. n = 5, **p < 0.01 versus control group; ^++^*p* < 0.01 for HG group versus HG plus tempol co-treatment group.

Hyperglycemia is a known inducer of reactive oxygen species and lipid peroxidation products such as 4-HNE. We next tested the effects of high glucose exposure on AMPKα2 activity in HUVECs. As shown in Fig. 3E and F, high glucose (HG, d-glucose 25 mM), but not the osmotic control l-glucose (19.5 mM L-glucose+5.5 mM d-glucose), significantly attenuated AMPKα2 activity. Although neither GSH nor tempol alone affected AMPKα2 activity (Fig. 3E and F), co-administration of either antioxidant with high glucose ablated the inhibitory effects of HG on AMPKα2 activity.

### 3.10 GSH and tempol disrupt the association between 4-HNE and AMPKα2

4-HNE can allosterically inhibit protein function by binding to residues essential for enzymatic activity. We therefore examined whether 4-HNE physically interacts with AMPKα2 in cultured HUVECs. Immunoprecipitation of 4-HNE-bound proteins with a 4-HNE-specific antibody revealed an increase in 4-HNE-modified proteins upon 4-HNE treatment (Fig. 4A). Probing these immunoprecipitates for AMPKα1 showed only a faint band in both vehicle - treated and 4-HNE-treated HUVECs (Fig. 4A), suggesting minimal modification of AMPKα1. Reciprocally, immunoprecipitation of AMPKα1 followed by 4-HNE immunoblotting yielded no detectable signal (Fig. 4B). In contrast, AMPKα2 immunoprecipitated from 4-HNE-treated HUVECs exhibited a clear 4-HNE-positive band, which increased in intensity from 5 to 30 min of treatment (Fig. 4C), conforming a time-dependent interaction between 4-HNE and AMPKα2.

GSH disrupted the 4-HNE-AMPKα2 interaction under both basal and high-glucose conditions (Fig. 4D-G). Similarly, tempol (100 μM) abolished the high glucose-enhanced interaction between 4-HNE and AMPKα2 (Fig. 4H, I).

### 3.11 Interaction between 4-HNE and AMPKα2 in human atherosclerotic plaques

**Fig. 5:**
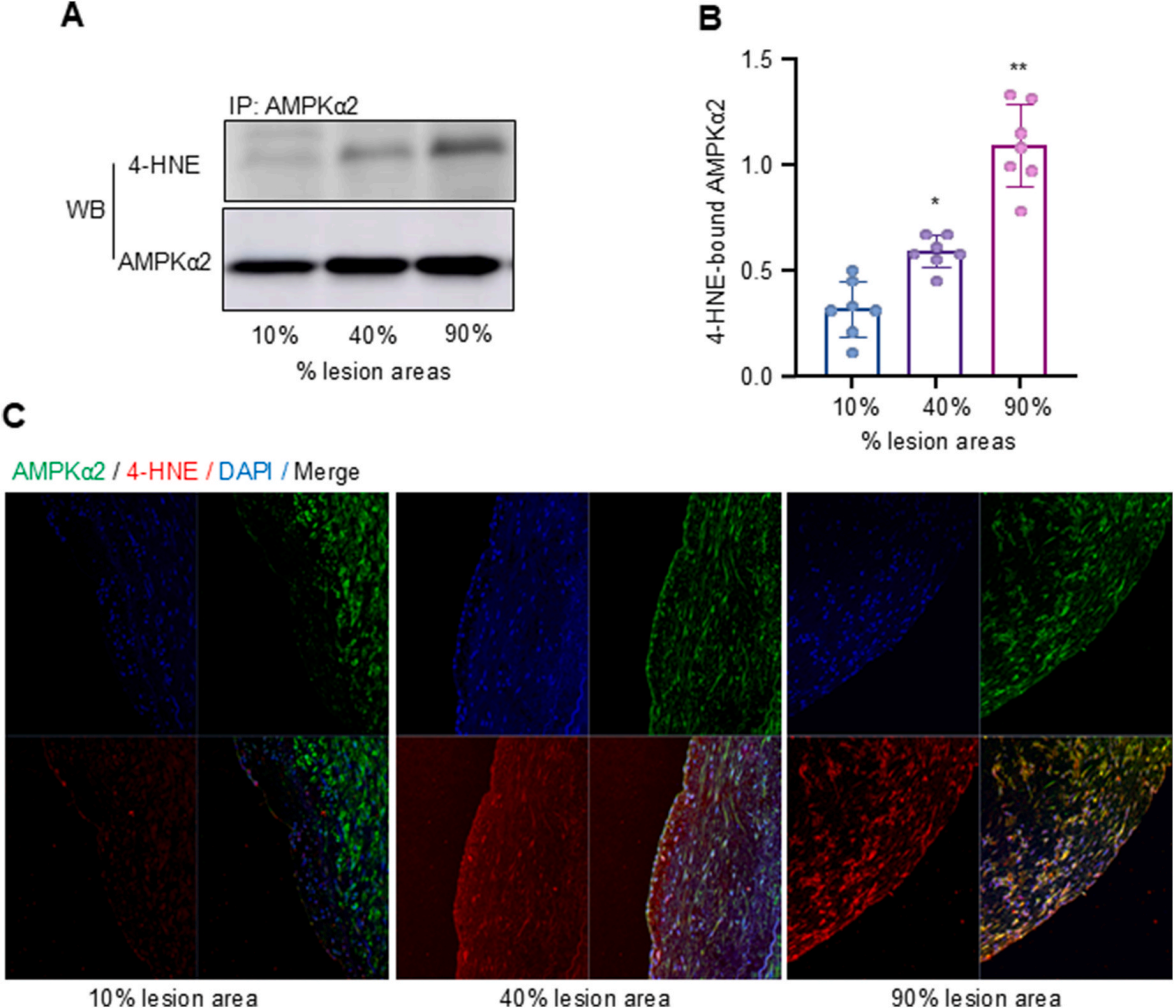
Molecular interaction between 4-HNE and AMPKα2 in human atherosclerotic lesions. (**A–B**) Immunoprecipitation verification and quantitative analysis of 4-HNE-bound AMPKα2 in human atherosclerotic tissues. Total protein lysates extracted from human atherosclerotic lesions were subjected to AMPKα2 immunoprecipitation, followed by immunoblotting using anti-4-HNE antibody or control IgG antibody. n = 7, *p < 0.05, **p < 0.01 versus the 10% lesion area group. (**C**) Immunofluorescence co-localization of 4-HNE and AMPK in human atherosclerotic lesions. Representative immunofluorescence staining was performed to label cell nuclei (blue), 4-HNE (red), and total AMPK protein (green) in human atherosclerotic lesion sections. Merged fluorescence images demonstrate substantial co-localization (yellow signals) of AMPK and 4-HNE within lesion tissues.

We examined 4-HNE–AMPKα2 binding in coronary atherosclerotic plaques with varying plaque coverage (10%, 40%, or 90% of the luminal surface). Co-immunoprecipitation revealed that 4-HNE–AMPKα2 binding increased with plaque severity (Fig. 5A and B). Double-label immunofluorescence showed that while AMPKα2 immunostaining was unaffected by plaque size, 4-HNE levels correlated with lesion severity and co-localized with AMPKα2 in advanced plaques (Fig. 5C).

### 3.12 Identifications of Cys174 and Cys200 as 4-HNE - bound sites in AMPKα2 by MALDI - TOF/TOF mass spectrometry

**Fig. 6:**
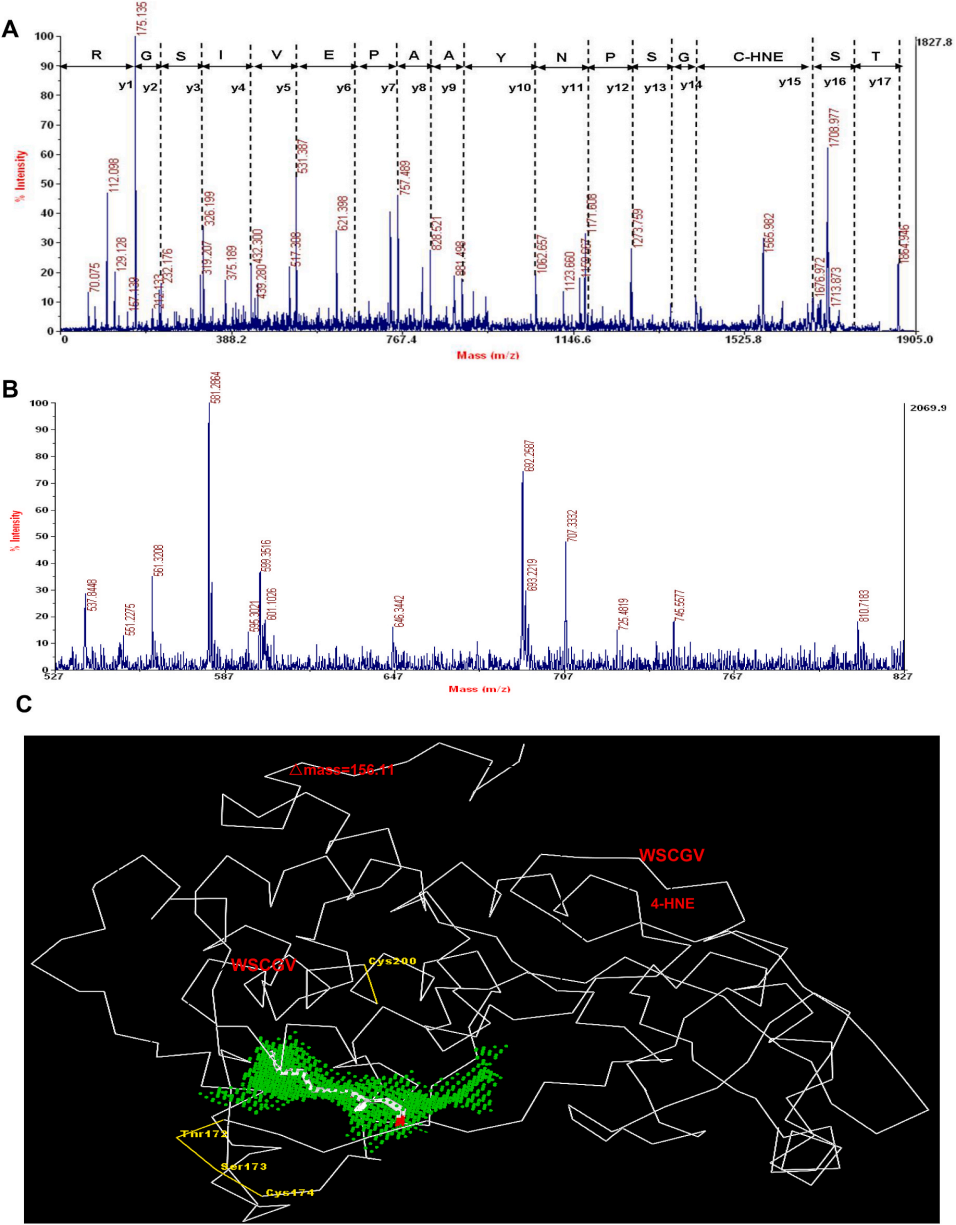
MALDI-TOF/TOF mass spectrometry and structural simulation analysis of 4-HNE-bound AMPKα2. (**A–B**) Mass spectrometric identification of 4-HNE modification sites on AMPKα2. MALDI-TOF/TOF mass spectrometry was performed to verify the covalent modification of AMPKα2 by 4-HNE. The peptide sequence is displayed as follow: Left - N-terminus; Right: C-terminus. y_1_ = R, y_2_ = R + G. Mass spectral results confirm specific 4-HNE conjugation at the C174 (A) and C200; (B) cysteine residues of AMPKα2. (**C**) Spatial structural simulation of 4-HNE–AMPKα2 binding interaction. Molecular structural simulation was conducted to characterize the spatial binding conformation and interaction mode between 4-HNE and the AMPKα2 protein.

Next, we used MALDI-TOF/TOF mass spectrometry to identify cysteine sites for 4-HNE. As depicted in Fig. 6A and B, MALDI-TOF/TOF analysis confirmed that 4-HNE covalently modified AMPKα2 at Cys174 and Cys200, as evidenced by an increase in peptide molecular mass corresponding to the mass of 4-HNE. Molecular modeling of the 4-HNE-AMPKα2 interaction, shown in Fig. 6C, indicated that Cys174 and Cys200 are both spatially close to Thr172, a critical phosphorylation and activation site in AMPK. It is likely that binding of 4-HNE to Cys174 and Cys200 inhibits AMPK phosphorylation at Thr172 and consequently suppress AMPK activity.

### 3.13 4-HNE inhibits AMPKα2 activity by modifying Cys174 and Cys200

**Fig. 7:**
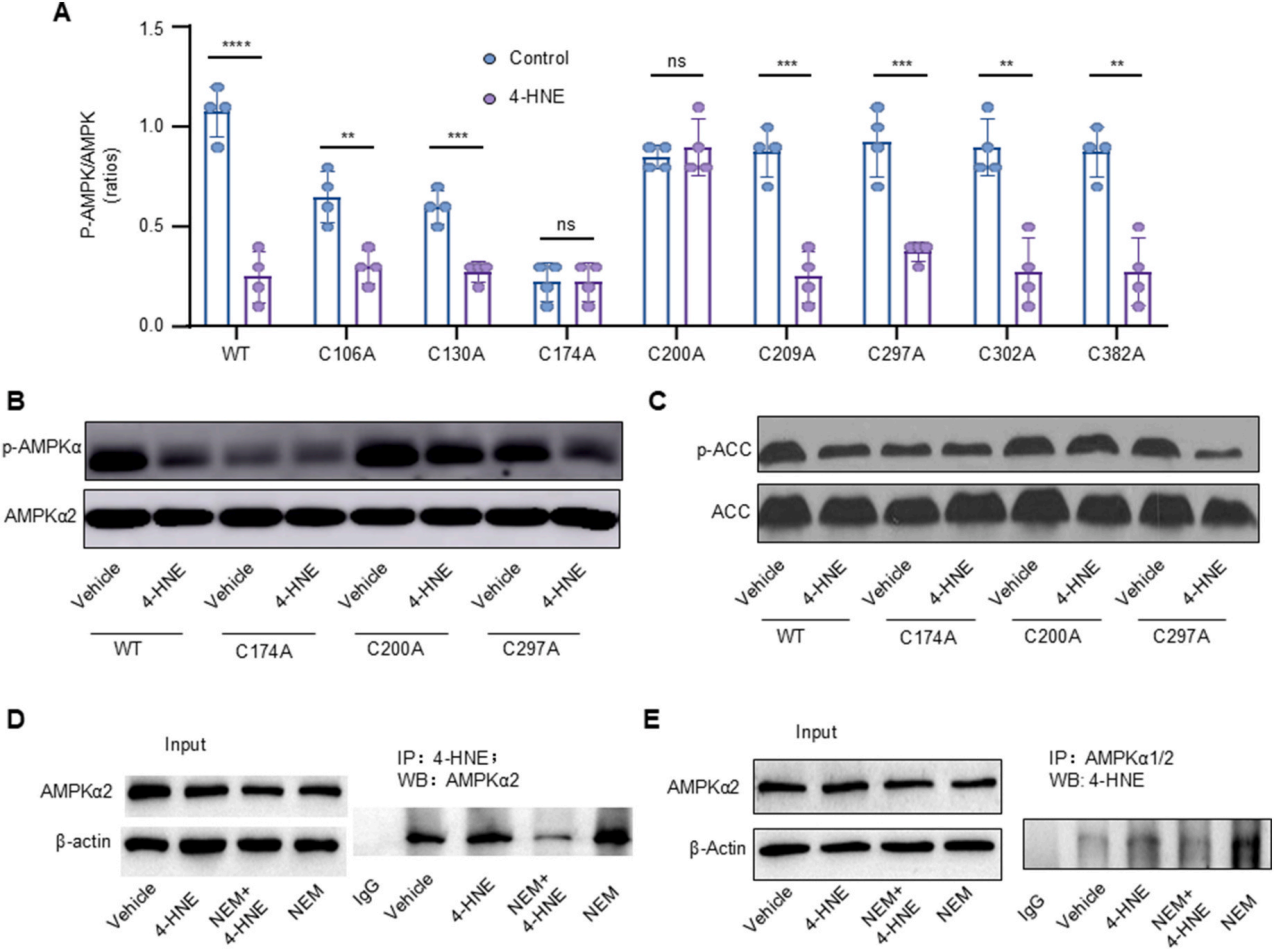
4-HNE suppresses AMPKα2 enzymatic activity via covalent modification of the C174 and C200 cysteine residues of AMPKα2. (**A**) AMPKα2 activity in HEK293 cells expressing wild-type or mutant AMPKα2 following 4-HNE stimulation. HEK293 cells were transfected with either wild-type (WT) AMPKα2 or AMPKα2 plasmids carrying specific cysteine-to-alanine (Cys-Ala) point mutations, followed by treatment with 10 μM 4-HNE for 24 h n = 4, **p < 0.01 versus the corresponding control groups. (**B**) Western blot detection of total AMPK and phosphorylated AMPK (Thr172) expression in 4-HNE-treated HUVECs. HUVECs were transfected with WT AMPKα2 or mutant AMPKα2 constructs for 24 h, and the transfected cells were subsequently exposed to 10 μM 4-HNE or vehicle control for an additional 24 h. Western blot analysis was performed to assess the protein levels of total AMPK and phosphorylated AMPK at the Thr172 site. The blot is a representative of three blots from three independent experiments. **(C)** Western blot detection of total ACC and phosphorylated ACC (Ser79) expression in 4-HNE-HUVECs. The blot is a representative of three blots from three independent experiments. (**D**) NEM abolishes the binding interaction between 4-HNE and AMPKα2 in HUVECs. HUVECs were pretreated with 25 μM NEM for 30 min prior to 10 μM 4-HNE administration. 4-HNE-positive proteins was first enriched through immunoprecipitation, and the immunoprecipitated products were then immunoblotted with specific antibodies against AMPKα2. All blotting results are representative of three independent biological replicates. (**E**) NEM abolishes the binding interaction between 4-HNE and AMPKα2 in H9c2 cells. H9c2 cells were subjected to 4-HNE treatment with or without NEM preconditioning. The endogenous expression levels of AMPKα1 and AMPKα2 were examined via Western blot analysis using their respective specific antibodies. AMPKα proteins was first enriched through immunoprecipitation, and the immunoprecipitated products were then immunoblotted with specific antibodies against 4-HNE. All blotting results are representative of three independent biological replicates.

To identify 4-HNE binding sites on AMPKα2, we assessed 4-HNE binding to AMPKα2 variants containing individual Cys-to -Ala substitutions. HEK293 cells were transfected with WT or mutant AMPKα2 plasmids and treated with or without 4-HNE. 4-HNE inhibited WT AMPKα2 and the C106A, C130A, C209A, C297A, C302A, and C382A mutants. In contrast, AMPKα2 activity was not inhibited in cells expressing the C174A or C200A mutants (Fig. 7A), indicating that these residues are critical for 4-HNE–mediated inhibition.

We further examined the effects of 4-HNE on WT, C174A, C200A, and C297A AMPKα2 in HUVECs. 4-HNE inhibited Thr172 phosphorylation of WT and C297A AMPKα2, as well as ACC phosphorylation at Ser79. In contrast, 4-HNE had no effect on AMPK or ACC phosphorylation in cells expressing C174A or C200A mutants (Fig. 7B and C). These findings indicate that Cys174 and Cys200 mediate AMPKα2 inhibition by 4-HNE, possibly by modification of these residues.

### 3.14 Thiol blocking with N-ethylmaleimide blocks 4-HNE binding to AMPKα2

4-HNE can form both Michael adducts (predominantly with cysteine) and Schiff bases (with lysine residues). To test the cysteine dependency of 4 - HNE - mediated AMPKα2 inhibition, we utilized N - ethylmaleimide (NEM), a thiol blocker. HUVECs or H9c2 cells were pretreated with NEM (25 μM) 30 min before adding 4–HNE for 30 min duration. NEM alone did not impact the protein levels of AMPKα2 (Fig. 7D). To assess NEM's effect on 4-HNE-enhanced binding to AMPKα2 in HUVECs, 4-HNE-positive proteins were immunoprecipitated from vehicle (DMSO)-treated, 4-HNE-treated, NEM-pretreated+4-HNE-treated, and NEM-treated HUVECs using an anti-4-HNE antibody. Subsequently, Western blot analysis with an anti-AMPKα2 antibody detected the AMPKα2 protein. As shown in Figs. 7D and 4-HNE significantly increased the levels of AMPKα2 in the immunoprecipitates with the 4-HNE-specific antibody. Moreover, when 4-HNE-positive proteins were immunoprecipitated from 4-HNE-treated HUVECs, pretreatment with NEM before adding 4-HNE abolished the 4-HNE-enhanced AMPKα2 levels. Notably, NEM alone led to a clear increase in 4-HNE-bound AMPKα2 in HUVECs.

Finally, in H9c2 cells, we examined NEM's effect on 4-HNE-AMPK binding. First, AMPKα2 was immunoprecipitated, followed by Western blot detection with an anti-4-HNE antibody. As depicted in Fig. 7E, neither 4-HNE nor NEM treatment alone changed the levels of AMPKα2 in H9c2 cells. We also immunoprecipitated AMPKα2 from vehicle-treated, 4-HNE-treated, NEM-pretreated+4-HNE-treated, and NEM-treated H9c2 cells using an anti-AMPKα2 antibody, and then detected 4-HNE-positive AMPKα2 protein via Western blot with an anti-4-HNE antibody. A weak band of 4-HNE-positive AMPKα2 was seen in vehicle-treated H9c2 cells. 4-HNE treatment significantly increased 4-HNE-positive AMPKα2. Additionally, pretreatment with NEM before 4-HNE exposure in H9c2 cells significantly reduced 4-HNE-positive AMPKα2 (Fig. 7E). Interestingly, NEM treatment alone also increased the detection of 4-HNE-bound AMPKα2 (Fig. 7E). Neither 4-HNE nor 4-HNE plus NEM treatments affected the levels of AMPKα1 (data not shown). Collectively, these results support a primary role for cysteine modification rather than Schiff base formation under 4-HNE exposure.

## 4 Discussion

In this study, we elucidated the effects of 4-HNE on AMPKα2 activity and our results demonstrated that 4-HNE modifies AMPKα2 both in vitro and in cultured HUVECs, leading to a time- and dose-dependent inhibition of AMPK activity. Notably, this inhibition occurs at concentrations (≤5 μM) that do not significantly affect cell viability, indicating that suppression of AMPK is unlikely to be a secondary consequence of cytotoxicity but instead reflects a selective biochemical effect. Further, we identified Cys174 and Cys200 as the pivotal residues mediating 4-HNE-dependent AMPKα2 inhibition. MS/MS analysis confirmed that 4-HNE treatment triggers covalent adduct formation at these two cysteine sites, accompanied by increased molecular weight consistent with residue modification. Site-directed mutagenesis further validated that individual substitution of Cys174 or Cys200 completely abolishes 4-HNE-induced suppression of AMPK phosphorylation and catalytic activity, whereas mutations at other cysteine residues exert no functional effect. In line with these biochemical observations, functional assays demonstrated that 4-HNE fails to inhibit AMPK activation and downstream ACC phosphorylation in cells expressing C174A or C200A AMPKα2 mutants. In parallel, the pretreatment of either HUVECs or H9c2 cells with NEM, a cysteine blocker, abolished the binding of 4-HNE and AMPKα2, supporting a primary role for cysteine modification rather than other reactions such as Schiff base formation under the conditions used. Structural modeling revealed that Cys174 and Cys200 are spatially proximal to Thr172, the canonical phosphorylation site required for full AMPKα2 activation, suggesting that covalent modification of these two cysteine residues may sterically or biochemically perturb Thr172 phosphorylation, thereby repressing AMPKα2 catalytic activity. The translational relevance of our findings is validated in the Akita type 1 diabetic mouse model, where elevated vascular oxidative stress correlates with reduced aortic AMPK activity. Notably, Tempol treatment restores vascular AMPK function without altering systemic metabolic homeostasis, indicating that oxidative stress, rather than hyperglycemia per se, is the primary mediator of *in vivo* AMPK suppression. Consistent with animal findings, human coronary plaque analysis revealed markedly repressed AMPK activation, characterized by reduced Thr172 phosphorylation with unaltered total AMPK expression. In human atherosclerotic lesions, elevated 4-HNE levels inversely correlate with AMPKα2 activity, and enhanced 4-HNE–AMPKα2 interaction positively associates with plaque severity. Collectively, these clinical and preclinical data solidify the causal link between lipid peroxidation and AMPK dysfunction in human atherosclerotic vascular disease.

The unique electrophilic properties of 4-HNE underpin its preferential reactivity toward protein cysteine thiols. As an electrophilic α,β-unsaturated aldehyde, 4-HNE readily undergoes Michael addition with nucleophilic thiol groups. Consistent with prior work, 4-HNE exhibits a well-established residue reactivity hierarchy (Cys ≫ His ≫ Lys) [33] with second-order rate constants ranging from 1000 to 10,000 M^−1^ s^−1^, enabling efficient covalent modification under physiological conditions. In accordance with this chemical behavior, GSH efficiently abrogate 4-HNE-triggered AMPKα2 modification in both recombinant proteins and intact cultured cells. Similarly, the antioxidant Tempol substantially rescues 4-HNE-mediated AMPK inhibition. These findings support a core redox-dependent mechanism wherein cysteine-specific covalent adduction drives AMPKα2 inactivation. Notably, the present data do not exclude potential auxiliary contributions from modifications at other amino acid residues.

Our mechanistic model is corroborated by previous independent studies. Shearn et al. [44]. reported that 4-HNE exposure induces AMPKα/β carbonylation and functional inhibition in hepatocytes, with LC-MS mapping verifying Cys174 as a major 4-HNE-modified residue. Broader literature also confirms that micromolar 4-HNE concentrations induce widespread protein adduction across multiple cellular contexts [[45], [46], [47], [48], [49], [50]]. As a major lipid peroxidation byproduct, 4-HNE is generated from polyunsaturated fatty acids enriched in LDL [46]. Multiple vascular and immune cell types involved in atherogenesis promote LDL oxidation and internalize 4-HNE-modified LDL, leading to selective 4-HNE accumulation in atherosclerotic lesions but not healthy arterial walls. Functionally, 4-HNE drives multiple pro-atherogenic events, including enhanced endothelial immune cell adhesion, endothelial apoptosis, barrier dysfunction, and vascular smooth muscle cell proliferation via mitogen-activated protein kinase signaling activation [[47], [48], [49], [50]]. In this study, 4-HNE colocalized with AMPKα in human atherosclerotic plaques, and this interaction correlated with reduced AMPK activity. Combined with our previous findings that AMPKα deficiency exacerbates endothelial dysfunction [32], neointimal formation, and atherosclerosis progression [51], the present results establish a robust molecular cascade linking 4-HNE accumulation, AMPKα2 suppression, and human atherogenesis.

4-HNE reacts rapidly with protein thiolate groups under physiological conditions, with rate constants supporting diffusion-limited adduct formation within milliseconds to seconds. This high chemical reactivity accounts for the robust protective effects of GSH, which completely eliminate AMPKα2 modification by both exogenous and endogenously generated 4-HNE. Tempol-mediated antioxidant intervention similarly rescues 4-HNE-induced AMPK suppression, further validating oxidative stress as a central upstream driver of AMPK dysfunction. As a primary cellular antioxidant, GSH confers vascular protection against 4-HNE toxicity. Pathological states including atherosclerosis and diabetes feature depleted intracellular GSH, rendering vascular tissues susceptible to exacerbated lipid peroxidation and oxidative injury [52,53]. Our data further demonstrate that GSH disrupts the physical interaction between 4-HNE and AMPKα2, thereby blocking subsequent AMPK inhibitory signaling.

High 4-HNE concentrations (>10 μM) are known to induce robust ROS overproduction, cellular apoptosis, and widespread non-specific protein modification, accompanied by dysregulation of upstream kinases including LKB1 and global cellular stress responses [[54], [55], [56]]. In contrast, the low, pathophysiologically relevant 4-HNE concentration (≤5 μM) used in this study avoids non-specific cytotoxicity and off-target signaling perturbations, enabling specific and precise characterization of the direct 4-HNE–AMPKα2 regulatory axis. In healthy humans, basal plasma 4-HNE levels range from 0.3 to 0.7 μM [57,58], rising to 1–20 μM [59] under oxidative stress-associated pathological conditions such as atherosclerosis. While free 4-HNE quantification in intact atherosclerotic plaques remains technically challenging, localized microdomains (e.g., lipid membranes and plaque cores) can achieve concentrations up to ∼100 μM [59]. Prior studies [[59], [60], [61]] investigating 4-HNE-induced LKB1 signaling dysregulation adopt 10–40 μM 4-HNE; by contrast, the low dosage (<10 μM) utilized here eliminates LKB1-related confounding effects and ensures the specificity of the observed AMPKα2 regulatory phenotype.

Antioxidant rescue experiments further corroborate our proposed mechanistic model. Both GSH and Tempol restore AMPKα2 Thr172 phosphorylation and disrupt 4-HNE–AMPKα2 binding, confirming that oxidative cysteine adduction is essential for 4-HNE-driven AMPKα2 inhibition. Given the diminished intracellular GSH abundance in atherosclerotic and diabetic vascular tissues [52], impaired endogenous antioxidant capacity represents a critical factor that sensitizes vascular cells to 4-HNE-mediated AMPKα2 dysregulation *in vivo*.

AMPKα2 is a central regulator of vascular redox homeostasis, whose activation suppresses oxidative stress and inflammatory signaling. Here, we demonstrate that 4-HNE-mediated covalent modification induces sustained AMPKα2 inactivation, uncovering a novel mechanism by which lipid peroxidation products disable endogenous vascular protective pathways. Although this study does not fully dissect downstream signaling cascades, the consistent inverse relationship between 4-HNE abundance and AMPKα2 activity across cellular, animal, and human tissue models supports a critical role for AMPKα2 in maintaining endothelial homeostasis. Specifically, this work defines an unprecedented redox regulatory mechanism linking oxidative lipid damage to vascular metabolic dysfunction: 4-HNE selectively modifies AMPKα2 at Cys174 and Cys200, triggering persistent AMPKα2 suppression. The ability of antioxidant intervention to restore AMPKα2 activity further suggests that targeting redox-dependent AMPKα2 modification holds translational therapeutic potential for oxidative stress-related vascular diseases, pending further clinical validation.

The translational significance of our findings is corroborated in the Akita type 1 diabetic mouse model. Here, elevated vascular oxidative stress is associated with reduced aortic AMPK activity. Remarkably, treatment with Tempol restores vascular AMPK function without disrupting systemic metabolic homeostasis. This indicates that oxidative stress, rather than hyperglycemia itself, is the primary mediator of *in vivo* AMPK suppression. Consistent with the animal-based findings, analysis of human coronary plaques reveals a marked repression of AMPK activation, characterized by reduced Thr172 phosphorylation despite unaltered total AMPK expression. In human atherosclerotic lesions, elevated 4-HNE levels exhibit an inverse correlation with AMPK activity, and an enhanced 4-HNE–AMPKα2 interaction positively correlates with plaque severity. Collectively, these clinical and pre-clinical data strengthen the causal connection between lipid peroxidation and AMPK dysfunction in human vascular disease.

Several study limitations warrant acknowledgment. First, while a robust association between 4-HNE adduction and AMPKα2 inhibition is established, definitive *in vivo* causality requires further genetic or pharmacological validation. Second, this study focuses on Cys174 and Cys200 as primary modification sites and cannot exclude potential contributions from uncharacterized cysteine residues or alternative post-translational modifications to AMPK regulation. Third, direct biophysical evidence is lacking to resolve the structural basis by which 4-HNE modification impairs Thr172 phosphorylation. Finally, human tissue analyses lack detailed documentation of sample demographics and patient heterogeneity, which may limit the generalizability of clinical observations.

In conclusion, our findings reveal that 4-HNE inhibits AMPKα2 activity through oxidative modification of specific cysteine residues. These results highlight the importance of AMPKα2 as a redox-sensitive modulator of vascular health and suggest that targeting oxidative stress pathways could offer a therapeutic strategy for diseases like atherosclerosis, where AMPK activity is compromised.

## Disclosures

None.

## Sources of funding

This work was supported in part by funding from the Nature Science Foundation of China (82530049 and 82470443 to M.H.Z.) and by Funded by Tianjin Key Medical Discipline Construction Project (TJYXZDXK-3-002C). Dr. Jie Deng's laboratory is supported in part by Shannxi Social Development Funding, China (Grant No: 2024SF2-GJHX-12).

## CRediT authorship contribution statement

**Shengyue Li:** Data curation, Formal analysis, Investigation, Methodology, Writing – original draft. **Xin Zhang:** Data curation, Investigation, Methodology. **Guocheng Zhang:** Formal analysis. **Huihui Yang:** Investigation, Methodology. **Jie Deng:** Resources. **Ming-Hui Zou:** Conceptualization, Formal analysis, Funding acquisition, Supervision, Writing – original draft, Writing – review & editing.

## Declaration of competing interest

The authors declare that they have no known competing financial interests or personal relationships that could have appeared to influence the work reported in this paper.
